# Reception of the values of the Aeschylus drama and mnemonic imprints by ancient tragedy spectators

**DOI:** 10.12688/openreseurope.15179.1

**Published:** 2022-11-03

**Authors:** Konstantinos Mastrothanasis, Theodore Grammatas

**Affiliations:** 1 Department of Primary Education, University of the Aegean, Rhodes, Greece; 2 Department of Primary Education, National and Kapodistrian University of Athens, Athens, Greece

**Keywords:** Greek tragedy, ancient drama, Aeschylus, Seven against Thebes, humanitarian values, theatrical codes, mnemonic imprints, reception of Ancient Greek Drama

## Abstract

**Background:** Ancient Greek tragedy remains today a special dramatic genre that expresses the concept of the classic through time, perhaps better than any other form of art and culture, representing, as a theatrical expression, the vision of the conception and expression of values of a particular era. In this context, the purpose of the present research is to study the humanitarian values of European culture, as they are expressed in ancient Greek drama, and to highlight the way in which these values are projected through modern drama and are impressed on the spectators.

**Methods:** To achieve this goal, 105 spectators watched the tragedy of Aeschylus ‘Seven against Thebes’ directed by Cesaris Grauzinis and answered, both immediately after watching the performance and six months later, a questionnaire, in order to record their opinions about the theatre performance they had attended.

**Results:** According to the findings of the comparative analyses, it emerged that the messages and values governing the work remain unchanged for its viewers over time. The memory is based on original audio-visual elements and directorial findings, confirming that it preserves the messages of the symbolism of the performance as well as the channels through which they were conveyed to the audience.

**Conclusions: **The correspondences between the past and the present, as well as the contrasts on stage, contributed to the reproduction of the fundamental moral values that the dramatic work brought, highlighting the work and messages of Aeschylus.

## Plain language summary

The aim of the present study is to investigate how humanitarian values are displayed in ancient Greek drama and how they are perceived by the modern viewer when watching an ancient tragedy performance. To achieve this, a group of spectators of ancient Greek drama was selected and they were involved in the particular research. After watching Aeschylus' tragedy ‘Seven against Thebes’, spectators responded in a questionnaire both immediately as well as six months later, in order to record their opinions about the performance they had watched. From the analysis of their responses in the two-time phases of the research, it emerged that the messages and values that govern the play remain unchanged for the viewer over time and that the audio-visual elements and the directorial findings of the performance help to convey the messages as well as symbolism of ancient Greek drama.

## Introduction

Ancient drama is considered to be a link between the classic and the modern, the present and the past. Apart from any aesthetic and artistic contexts through which it forms and communicates with the modern audience through the stage as a theatrical performance, its values represent the diachronic and the universal in cultural creation ( Grammatas, 2015; Kladaki, 2010b; Patsalidis, 2022). The ancient drama represents the vision of the conception and expression of values in a specific historical space and time, which are still encountered today, while defining the culture, the ideals, and the ideals of mankind ( Adkins, 1982; Grammatas, 2012; Kladaki, 2010a; Papadopoulos, 2021).

The insinuate and allegorical reason with which the heroes approach violence, power, ambition, personal responsibility for situations, respect for the gods, etc. creates a distance between the ancient tragedy and the goal of an accurate representation of historical events and persons, but approaches and realizes the vision of the conception and expression of the universal, the collective and the diachronic ( Grammatas & Dimaki-Zora, 2018; Kladaki, 2009; Kladaki, 2011). Therefore, it can always highlight issues such as the impasse of violence, arrogance, the absurdity and futility of war, the vanity and arrogance of the powerful, the morality of the weak, and highlight values such as freedom, democracy, peace, human dignity, dialogue, and human rights ( Grammatas, 2022).

In the context of the program European Union's Horizon 2020 (No 101004949) entitled ‘Values Across Space and Time’ (VAST) ( https://www.vast-project.eu/) ( Berni *et al.,* 2021, pp. 82–83), the participation of the National and Kapodistrian University of Athens was made in order to study and highlight the humanitarian values of European culture, as expressed in Ancient Greek Drama and Theater and the way in which they are projected through contemporary drama, with special emphasis on those that are considered fundamental to the European Union. Our aim is to examine how these values are revisited in the present through modern theatrical reproduction of the classical plays. We will analyse how the contemporary appropriations of the texts create new meanings through acting, music, voice, sets, etc. and how a director chooses to present values coming from antiquity to the modern audience.

Based on a first empirical audience study on the Aeschylus's ‘Seven against Thebes’ tragedy ( Torrance, 2007), directed by Cezaris Grauzinis ( National Theatre of Northern Greece, 2017), which received warm reviews, it became clear that viewers not only perceive, but also embrace a variety of well-established timeless moral values of ancient drama, as they were rendered to today through the modern gaze of the director ( Mastrothanasis & Papakosta, 2021). This performance was chosen as a case study because of the number of topics it presents to its spectator. The ‘Seven against Thebes’ by Aeschylus is the second oldest surviving tragedy to date, first presented in Athens in 467 BC. In it Polynices, son of Jocasta and Oedipus, leaves Thebes after refusing to hand over power to his brother, Eteocles. Keeping the agreement that it would be shared alternately, he returns to claim it on terms of battle, having as an alliance the king of Argos, Adrastos, for organising a campaign against Thebes. Seven commanders from each of the two opposing troops line up on either side of the seven gates of the city. Eteocles sends to the six gates of Thebes six generals to defend the city and protect the people from slavery, while he goes to defend himself the seventh gate, the one to which Polynices arrives. The outcome of the siege is good for the city, Thebes is saved from its enemies, but the two brothers Polynices and Eteoklis kill each other, implying that the generation of Laios is lost for good ( Anderson, 2015; Brown, 1977; Cameron, 1971; Dowden, 2016; Stehle, 2005; Torrance, 2007; Wong, 2022, pp. 281–306).

In the first empirical audience study of Mastrothanasis and Papakosta (2021) which was initially conducted in the early summer of 2021 and was completed at the end of June of the same year, involved 105 people who watched the show online due to the emergency conditions created by the COVID-19 pandemic ( Karantzouli, 2022; Liedke & Pietrzak-Franger, 2021; Timplalexi, 2020). From the findings that emerged from the investigation and study of their views immediately after watching the play, it was found that it successfully projected through its theatrical codes the conceptual, the value and the archetypal content of Aeschylus' work, bringing to the surface a number of moral values / intellectual and political / social content expressed by the characters and the dramatic text, such as the hardships of a civil war, the value of human life and the need for reconciliation. Other important values and rights that the performance highlighted, for the viewers, were not only the respect for the dead and the universal right to burial, the devastating effects of power and ambition, but also the self-restraint of the people as an important advantage in managing difficult political situations and crises ( Mastrothanasis & Papakosta, 2021). The purpose of this study is to complete and expand the first empirical audience study through a comparative analysis of the findings of its first and second phase.

## Methods

### Purpose of the research

For a further study and thorough reading of the reception and reception of the values of the specific work (and through it of the ancient Greek tragedy), a new study of the same performance was designed and implemented, after a sufficient period of time since the first one. Its aim was to determine whether and to what extent in the consciousness of the spectators the mnemonic recordings and imprints from their communication with the valuable world of the work remain equally strong, or they have been inactivated due to the passage of time and the attenuation of the intense memories that were challenged by the show as a ‘vehicle of importance’. In other words, to show whether and to what extent the visual and audio-visual framing of the text, and therefore of the values that emanate from it, remains active in the consciousness of the spectators or weakens due to the reduced memorization of the stage spectacle.

In the first case, the acceptance and ascertainment of the universality and timelessness of the messages of the tragedy would be confirmed, putting the author himself and his tragic speech at the forefront, while in the second, it would somewhat limit the general acceptance with a corresponding adoption of the play, as an undisputed carrier of the message, replacing the author by the director. The first empirical research was followed by the second one, which is presented and comes to give answers to how the values recorded in the text (through the representation) work and are recorded in the viewer's consciousness over time, through a comparison between their views in two different time periods.

### Ethical statement

During the research design, care was taken so that it was in line with the provisions and guidelines of the rules of research ethics and ethics as they are reflected internationally. The protocol was approved by the Institutional Review Board of the National and Kapodistrian University of Athens vide No. 17132, dated 34674/11-11-2020. Written informed consent for participating in the research was obtained from the participants before the beginning of the study which referred to issues of informed consent, maintaining anonymity, confidentiality and safeguarding the privacy of personal data. It also guaranteed the protection of those who participated in it from possible exposure to suffering, psychological or physical danger or other negative effects. Participation in the study was voluntary and participants had the option to withdraw at any stage.

### Participants

The study involved 105 individuals who formed a non-random, but characteristically targeted sample, through purposive sampling ( Bryman, 2016; Hall & Roussel, 2017; Mason, 2017; Robinson, 2014). This sample size was considered satisfactory in relation to the purpose of the study in terms of theoretical saturation ( Glaser & Strauss, 2017; Hennink & Kaiser, 2022; Mastrothanasis & Kladaki, 2021, p. 155). The individuals were selected after a public call for expression of interest by the scientific head of the study. People over the age of 18 with a love and interest in watching ancient tragedy performances, after being informed about the study, could participate in its activities by electronically expressing an interest in participation. An attempt was made to include as many participants as possible in the long-term study given the risk of dropout that may have occurred due to the pandemic. We were interested in the participation of people who would watch a specific theatrical performance of ancient drama and immediately after watching it (a phase of study), as well as six months later (b phase of study), they would complete a specific questionnaire to record their impression and their views on its content and the messages it conveys to them. Each participant who expressed interest received a unique code, in order to anonymously manage their data at the various stages of the research and their comparative analysis in the two-time phases.

### Research design

To achieve the purpose of the research, the participants in the study electronically watched the tragedy ‘Seven against Thebes’ by Aeschylus in June 2021, which concerned the clash of Oedipus’s two sons for the throne, directed by Lithuanian director Cesaris Grauzinis, and translated by George Blanas, which was presented by the National Theater of Northern Greece, moved the audience, and received warm reviews. The theatrical performance was viewed electronically, at a time and a place convenient for the participants and not with a physical presence, due to the emergency conditions created by the COVID-19 pandemic and the SARS-Cov-2 virus, and to give the opportunity for people from different parts of Greece to participate in the study and express their opinion, since our concern was to include a more representative sample of adults as possible, with different demographic characteristics and cultural profile.

Immediately after the digital viewing of the theatre performance, the study participants filled in a questionnaire of open and closed questions in the Greek language, created in terms of the research’s subject and aim. The general purpose of completing the questionnaire was to record the opinion and investigate the opinions of the participants regarding the performance they had watched. Initially, the identification and evaluation of the main ideas and values of the work as they were given by the author in the original text and the way in which the viewers perceived them, through the theatrical performance. Its more specific objectives were not only to investigate the director's perspective on the actions of the show's protagonists, but also to formulate the viewers' opinions on conveying the play's messages to a modern audience.

For the construction of the questionnaire, initially, a set of questions was formulated in the Greek language regarding the specific theatre performance by the second of the authors of the article. These questions were independently evaluated for content and language by three experts who acted as the article’s peer reviewers. Together, they made mutual language modifications for the purpose of its optimal understanding and settled on its final version, which was distributed electronically to the study participants through a platform created for this purpose ( https://www.vast-project.eu/research-surveys/). The same questionnaire was also answered electronically six months later by the same viewers (December 2021-January 2022), after they were informed about it.

The data obtained from these questionnaires of both the first and the second phase were qualitatively analysed through thematic analysis according to Charmaz's constructivist approach ( Charmaz, 2014). This approach focuses on the subjective importance that participants attach to the expressed beliefs and values of researchers and was achieved based on the constant comparative method which was completed with the saturation of Glaser and Strauss (1967, p. 65) categories. The coding of the data included three phases a) that of open coding, b) that of axial coding and c) that of selective coding. Next, the core of the phenomenon under study was identified and all the higher order categories (general impressions and impact on memory, direction, theatrical codes, and symbols, values expressed by the roles of the performance) were connected to it, to complete the analysis with the development of a documented narrative ( Mastrothanasis & Kladaki, 2021, p. 155). The quantitative data obtained from the qualitative analysis were used for the presentation and comparison of the results through the use of their reference frequencies (N) and the relative frequency (%). Indicative verbal references were also utilized.

## Results

Of the total participants who participated in the first phase of the study, 76.2% were women and 23.8% were men, with a mean age of 42.2 ± 10.6 years (min=23, max=74). They had mainly studied in the field of humanities and had a high level of studies. The second phase of the study involved 55 of the 105 people who initially participated, while maintaining the proportion of characteristics they had during the first phase. 85% of the participants in the second phase were women and 15% men, with a mean age of 41.98 ± 11.2 years (min=23, max=74) (see Table 1).

**Table 1.** Frequencies (N) and percentages (%) of the demographic or other characteristics of the participants in the two phases of the study.

Characteristics	Categories	Phase A		Phase B	
		N	%	N	%
Gender	Male	25	23.8 %	8	15 %
	Female	80	76.2 %	47	85 %
Studies	Bachelor’s Degree	31	29.5 %	16	29 %
	Master	42	40.0 %	25	45 %
	Ph.D.	32	30.5 %	14	25 %
Subject of studies	Humanities	82	78.1 %	44	80 %
	Science	12	11.4 %	2	4 %
	Legal/social sciences	6	5.7 %	5	9 %
	Medical/nursing sciences	3	2.9 %	3	5 %
	Economics	2	1.9 %	1	2 %

### General impressions and impact on memory

According to the responses of the viewers, Grauzinis' direction received mostly positive reviews, both immediately after watching the performance (N = 47, 77.1% of the reports), and after a period of six months when the viewers were asked again (N = 19, 40% of the reports), since the impressions it left as a performance were excellent. As characteristically stated by one of the participants “It was a decent show that I watched with great interest and emotion” (participant 30) with the specific direction:

“…respecting the basic structure of the original text and presenting the facts that determine the structural axes of Aeschylus' work. At the same time, it proceeds to original stage combinations and succeeds in giving a more complex interpretation of Aeschylus values” (participant 25).

Viewers remember a lot of the elements of the show, six months after watching it and fragmentary scenes of the play (N = 28, 59% of reports), despite the passage of time. An indicative report of a viewer who, six months after watching the show, noted:

“I recalled the duel between Eteocles and Polynices. The violent way in which they were hugged, pushed away, and hugged again in order to be led to death. In addition, the director's costume choices, which highlighted his intention to give a modern look to the work, the simple setting, and the director's choice to include various multicultural elements in the work, gave the direction an innovative, unconventional character which does not depart from the word of Aeschylus” (participant 33).

Regarding the elements of the show that impressed the audience the most, it turned out that immediately after watching the show, the acting of the actors (N = 68, 38% of the reports) and the direction (N = 51, 28% of the reports) were the ones that impressed more. During the second phase of the study, the direction (N = 30, 31% of the reports) and the acting of the actors (N = 26, 27% of the reports) were the ones that were mentioned the most by the viewers, along with the elements from the visual context of the performance (N = 26, 27% of the reports) (See Table 2).

**Table 2.** Frequencies and percentages of reports to theatrical codes that impressed the viewers.

	Phase A		Phase B	
	Ν	%	Ν	%
Acting	68	38%	26	27%
Direction	51	28%	30	31%
Sound	37	21%	16	16%
Artistic frame ^1^	20	11%	26	27%
Text	4	2%	0	0%

^1 ^The category of visual frame includes the subcategories: Stage objects, stage space, lighting, and clothes.

On a more general basis, viewers believe that Aeschylus' dramatic text "deals with values and raises issues that concern the Greek and the European citizen nowadays" (N = 95, 90.5% of the reports of the first phase, N = 42, 95% of the reports in the second phase), as well as that they find "similarities and correspondences between the authoritarian discourse of the protagonists of the project and policies of today" (N = 78, 74.2% of the reports of the first phase, N = 46, 84% of reports in the second phase), creating reflection in the viewers. As one spectator characteristically stated:

“The show made me think about human nature. How man invents a particular metaphysics to legitimize his power over others. A feeling of futility towards anything temporary. Finally, the invention of the Other as an enemy, whether the Other is inside the walls or outside them” (participant 101).

Regarding the degree to which this particular performance creates the desire of the viewer to refer again to the text of Aeschylus, either reading it or watching another performance in the future, most of the respondents answered positively, both during the first phase of the study (N = 85, 81% of the reports), as well as during the second one (N = 33, 60% of the reports).

### Direction, theatrical codes, and symbols

The performance used a variety of stage objects, chosen by the director, with the main the stairs used by the two brothers, Eteocles and Polynices and the seven shields which were the basic equipment of the warlords (tent of seven shields). According to the spectators, the use of the stairs on stage by Eteocles and Polynices symbolize for the spectators mainly "the rise and fall of power" (N = 80, 38% of the reports of the first phase, N = 51, 32% of reports in the second phase). At the same time, the directing of the scene of the seven shields, with the selection of the warlords from the on-stage Chorus and their equipment is judged as a very original and functional scene which "introduces an ironic comedy that intensifies the tragedy of the situations" (N = 62, 46% of the reports of the first phase, N = 22, 33% of the reports in the second phase) and as an element that "animates the static and verbal action" (N = 56, 41% of the reports of the first phase, N = 30, 45% of reports in the second phase). In another scene, the last one, Chorus is unable to walk and move on stage, while Antigone and Ismene retreat with the corpses of their two brothers. This directorial manipulation marks the trapping of Chorus because of the choices and actions of his leaders (N = 69, 41% of the reports of the first phase, N = 34, 45% of the reports of the second phase).

In addition to the above, the viewers consider that the specific work of Aeschylus bequeaths to us universal positions and important messages such as the "skill of a civil war" (N = 99, 39% of the reports of the first phase, N = 49, 40% of the reports in the second phase), the "value of human life" (N = 61, 24% of the reports of the first phase, N = 26, 21% of the reports in the second phase) and the "need for a change of persons in power" (N = 33, 13% of the reports of the first phase, N = 21, 17% of the reports of the second phase). In fact, the direction of this play helped to "show the sufferings of war and the need for reconciliation" (N = 94, 89.5% of reports in the first phase, N = 44, 80% of reports in the second phase), while and in general the performance highlighted the conceptual / value content of Aeschylus' work (N = 91, 86.7% of the reports of the first phase, N = 34, 87% of the reports of the second phase).

Regarding the opinion of the spectators as to which of the visual-audio elements of the show emphasized the tragedy of the situations, during the first phase of the study, the most prevalent reports were a) lighting (N = 60, 21.7% of the reports of the first phase , N = 32, 19.6% of the reports in the second phase), b) the costume and scenographic choices (N = 59, 21.3% of the reports of the first phase, N = 36, 22.1% of the reports in the second phase) and c ) the evocative background music (N = 42, 15.2% of the reports of the first phase, N = 28, 17.2% of the reports of the second phase) (See Table 3).

**Table 3.** Frequencies and percentages of visual / audio reports highlighting tragedy per study phase.

	Phase A		Phase B	
	Ν	%	Ν	%
Lighting	60	21.7%	32	19.6%
The costume and scenographic choices	59	21.3%	36	22.1%
The evocative background music	42	15.2%	28	17.2%
The selection of the mixed Chorus and its overall management	40	14.4%	25	15.3%
Acting of actors	36	13.0%	22	13.5%
The on-stage activation of the mute Polynices and his kinesiological confrontation with Eteocles	40	14.4%	20	12.3%
Total	277	100%	163	100%

Of course, important in highlighting the tragedy was the selection of the mixed Chorus and its overall management (N = 40, 14.4% of the reports of the first phase, N = 22, 13.5% of the reports of the second phase), the acting of the actors (N = 36, 13% of the reports of the first phase, N = 22, 13.5% of the reports in the second phase), but also the on-stage activation of the mute Polynices and his kinesiological confrontation with Eteocles (N = 40, 14.4% of reports of the first phase, N = 20, 12.3% of reports in the second phase).

### Values expressed by the roles of the performance

Regarding the values of moral / intellectual and political / social content expressed by the roles of the performance during the first phase of the research, they are presented in the following table, in relative percentage frequency (%), sorted in descending order of importance. It follows that the most important values that are expressed are: a) equality and equality of women with men (gender equality), b) respect for the other person's personality, c) dialogue as a feature of democracy and d) the right reason and dialogue as a means of resolving disputes (See Table 4).

**Table 4.** Classified values based on percentage mode (Phase A).

Classification of Values	1	2	3	4	5	6	7	8	9	10
Gender equality	*19%*	11%	9%	6%	11%	12%	6%	10%	8%	8%
Respect for the other	8%	*18%*	11%	9%	12%	10%	16%	7%	4%	5%
Dialogue as a feature of democracy	7%	15%	15%	7%	*17%*	11%	8%	10%	8%	2%
The right, as a means of resolving disputes	11%	13%	8%	14%	8%	*15%*	10%	10%	6%	5%
The value and uniqueness of human life	10%	12%	9%	8%	6%	8%	*16%*	10%	10%	11%
The prudence as a way of life	6%	14%	16%	6%	11%	5%	7%	*18%*	11%	6%
Discipline and self-control of the people as important advantages in managing difficult political situations	13%	7%	3%	3%	10%	4%	12%	*21%*	13%	13%
Respect for the gods and faith in their help as a saving solution in managing difficult political situations and crises	10%	10%	10%	5%	3%	6%	9%	14%	*20%*	14%
The catastrophic effects of power obsession and ambition	12%	7%	5%	3%	5%	6%	10%	15%	*20%*	17%
Respect for the dead and the universal right to burial	16%	9%	4%	5%	3%	4%	7%	10%	*26%*	17%

During the second phase of the study, six months after watching the play, it was revealed that the classification of values by its viewers did not undergo significant changes (See Table 5). And in this case the most important values expressed by the persons of the project, based on a percentage mode, are:

**Table 5.** Classified values based on percentage mode (Phase B).

Classification of values	1	2	3	4	5	6	7	8	9	10
Gender equality	13%	*23%*	11%	9%	15%	4%	6%	8%	4%	8%
Respect for the other	6%	11%	9%	*17%*	15%	13%	6%	9%	11%	2%
Dialogue as a feature of democracy	2%	9%	13%	8%	*17%*	9%	15%	9%	13%	4%
The right, as a means of resolving disputes	4%	7%	6%	15%	15%	*17%*	13%	11%	11%	2%
The value and uniqueness of human life	4%	9%	13%	11%	13%	*15%*	6%	8%	9%	11%
The prudence as a way of life	4%	8%	9%	13%	9%	11%	11%	9%	*15%*	9%
Discipline and self-control of the people as important advantages in managing difficult political situations	7%	7%	11%	4%	5%	9%	13%	*22%*	9%	13%
Respect for the gods and faith in their help as a saving solution in managing difficult political situations and crises	2%	6%	8%	6%	15%	0%	15%	*25%*	15%	9%
The catastrophic effects of power obsession and ambition	13%	4%	8%	0%	2%	4%	4%	15%	*34%*	17%
Respect for the dead and the universal right to burial	2%	6%	8%	6%	15%	0%	15%	*25%*	15%	9%

a) the equality and equality of women with men (gender equality),

b) respect for the personality of the other,

c) dialogue as a feature of democracy and

d) right reason and dialogue as a means of resolving disputes.

As it turns out, the most important values expressed by the characters are constant in time for the viewers.

A key figure in the play is Eteocles, who can be approached on a case-by-case basis in the way he is presented, and the values expressed through his face. Regarding how the viewers impress the main elements of his appearance and behaviour on stage as a protagonist, in the prologue part of the play (initial monologue to the silent audience of warriors, dialogue with the messenger) and in his first interactive confrontation with Chorus, he appears in the memory of the spectators during the first phase of the study mainly as "disparaging towards women and their reactions" (N = 35, 33% of reports), "pious towards the gods, although he opposes his begging action Chorus "(N = 26, 25% of reports) and "Dynamic leader trying to take control of the city" (N = 25, 24% of reports). During the second phase of the study, six months later, viewers remember him again mainly as "a dynamic leader trying to take control of the city" (N = 25, 25% of reports), "derogatory towards women and their reactions" (N = 9, 16% of the reports) and "pious towards the gods, although he opposes the begging action of Chorus" (N = 7, 13% of the reports), highlighting from the comparative study of the reports that he is a leader who wanted to have complete control of his city with elements of contempt for women.

Regarding his final decision to confront his brother Polynices at the seventh gate, the spectators characterize his act mainly as a "combination of the fatal check from the divine will and the free will of the hero" (N = 70, 67% of reports of the first phase, N = 30, 55% of the reports in the second phase), with the confrontation of the two brothers (fratricide) being captured on stage as a necessary act, a consequence of the tragic destiny (N = 63, 60% of the reports first phase, N = 30, 55% of reports in the second phase). In fact, the stage presentation of Polynices worked positively in further recording the fraternal hatred (N = 83, 79% of the reports of the first phase, N = 45, 82% of the reports of the second phase). In his dialogue with the women of Chorus, who begged the gods to save them, Eteocles opposed the need for discipline, which he called as “the mother of victory”, thus contrasting "the fatalistic attitude of women towards the active and dynamic attitude of men." (N = 97, 92.4% of the reports of the first phase, N = 49, 89% of the reports of the second phase), with the relations between the two sexes being presented, generally unequal and discriminatory (N = 81, 77.1% of the reports of the first phase, N = 45, 82% of the reports in the second phase).

## Discussion

Based on the results of the comparative study of the data from the first and second phases of the research, this particular performance of Grauzinis received positive reviews even six months after watching it, while the majority of participants remembered many of its elements, despite the passage of time. Several referred to powerfully staged scenes that stood out in their minds or expressed thoughts about the show's value content that involved correspondences between past and present events. With this as a given, it seems that the stage spectacle still remains active in the public's consciousness and is a point of immediate reference and recall whenever the specific performance is mentioned for some reason ( Mastrothanasis, 2021; Papakosta *et al.*, 2020).

Regarding the value of content, the comparative analysis between the responses of the participants revealed a stability in terms of the impression and projection of fundamental timeless values through the play, such as that of gender equality, respect for the personality of the other and of dialogue as a key feature of democracy and a means of resolving disputes. The participants have strongly retained in their minds, six months after watching the performance, that it adequately highlighted the conceptual and value content of Aeschylus' work. Other values impressed upon the passage of time that the play deals with and bequeaths to the viewers are, the value of human life, as it is contrasted with the sufferings brought about by a civil war and the change of people in power. According to the participants of the study, these are values that largely concern the Greek and European citizen today. Political connections are also observed between the authoritative speech of the play's protagonists and today's politicians. Based on our findings, it is reasonable to conclude that the values and messages of the play, which are the defining elements of the specific work, are not only not marginalized among the total memory of the viewers, but still maintain their primary character, just as during the first their contact with the performance. This finding is in agreement with the existing literature in the field ( Grammatas, 2012; Mastrothanasis & Geladari, 2009).

The specific direction, based on the opinions highlighted by the study, respects the basic structure of the original text and presents the events that define the structural axes of Aeschylus' work, utilizing in a special way theatrical codes that are fixed in the viewer's memory ( Mastrothanasis, 2021; Mazzaro, 1984; Papakosta *et al.,* 2020). Elements that impressed and are remembered most by the viewers concerned the direction, the acting of the actors and elements from the visual context of the show. Viewers remember original scenographic and costume choices, the evocative background music, and functional elements of lighting in enhancing the tragedy of the situations emerging in the stage action. The use of music during the performance contributed to the further depiction of fraternal hatred, as did the stage presence of Polynices.

The direction appeared to satisfactorily project the sufferings of war and the need for reconciliation, the faces highlighted the interracial stereotypes, while original props such as the stairs or the seven shields, always according to the viewers' point of view, etched their memory. Memory has been confirmed to record, store and preserve the messages of the show's symbolism as well as the channels through which the audience conveyed them. For example, participants remembered that the stairs, a spectacular directorial invention of Grausinis that dominated the scene, symbolized the rise and fall of power, or that the director's handling of the scene of seven shields introduced an ironic comedy that heightened the tragedy of the situations, bringing static and verbal action to life. Another typical example is found in the presentation of Eteocles in the scene where the viewers have combined in their memory his presentation with elements of contempt for women and their unequal treatment.

All of the above prompts us to argue that the on-stage confrontation between fundamental humanistic values and acts of individualism, or elements of a modern versus traditional mentality and behaviour (e.g., devaluing women, etc.), they contribute to the preservation and reproduction of the fundamental moral values through the ancient drama, regardless of the stage mode chosen each time by the director. It is also worth noting that strong mnemonic traces and valuable imprints are left by strong directorial findings, such as those in the last scene of the drama. An example is the case of the Chorus, who was unable to walk or move on stage, as Antigone and Ismene withdrew with the corpses of the two brothers. It was confirmed that the participants strongly retained the elements of the particular theatre scene in their memory, as well as its meaning, that is, the entrapment of the Chorus due to the choices and actions of its leaders.

### Strengths and limitations of the study

Our longitudinal study was the first to explore the humanitarian values of European culture, as they are expressed in ancient Greek drama, and to highlight the way in which these values are projected through modern drama and are impressed on the spectators. One of the strengths of the study was that the research used a large number of ancient tragedy viewers from various regions of Greece, despite the limitations imposed by the emergency conditions created by the COVID-19 pandemic, allowing a more complete picture for the subject under study.

However, a limitation of the study arose from the digital viewing of the theatre performance related to the very nature of the way of viewing. Even though digital viewing allowed the participation of people from all over the Greek territory, its digital projection affected the reception and transmission of the messages of the theatrical performance. It is possible that the findings of the study would have been different if the performance had been watched in person, since messages would have been transmitted more effectively.

## Conclusions

In conclusion, from the analysis of the findings in the second phase, compared to those of the first, we can state that no substantial changes are observed in the responses of the participants. The messages and values that govern the work remain unchanged for its viewers. The memory continues to rely on original audio-visual elements and direction, confirming that it preserves the messages of the show's symbolism as well as the channels through which these were conveyed to the audience. However, the imprinting of the essential messages of the work on the consciousness of the spectators remains constant, which leads us to the conclusion to support that the values in ancient tragedy function independently of their stage setting, that is, of the specific performance of the work according to the point of view of director. The correspondences between the past and the present, as well as the contrasts on stage, contributed to the reproduction of the fundamental moral values that the dramatic work brought, highlighting the work and messages of Aeschylus.

## Data Availability

Zenodo: Reception of the values of the Aeschylus drama and mnemonic imprints by ancient tragedy spectators [Data set]. https://doi.org/10.5281/zenodo.7213455 ( Mastrothanasis & Grammatas, 2022). This project contains the following underlying data: Press data vast final.xlsx (Qualitative and quantitative data from VAST programme in original Greek) Press data vast final English version.xlsx (Qualitative and quantitative data from VAST programme in English) This project contains the following extended data: Questionnaire in English and Greek language. Data are available under the terms of a Creative Commons Attribution 4.0 International license (CC BY 4.0).

## References

[ref-40] Aeschylus (Writer) Grauzinis C (Director) Blanas G (Trans.) : Seven against Thebes. Live performance in National Theatre of Northern Greece, Thessaloniki.2017. Reference Source

[ref-1] Adkins AWH : Divine and Human Values in Aeschylus’ Seven against Thebes. *Antike Und Abendland.* 1982;28(1):32–68. 10.1515/9783110241402.32

[ref-2] Anderson AS : The Seven against Thebes at Eleusis. *Ill Classic Stud.* 2015;40(2):297–318. 10.5406/illiclasstud.40.2.0297

[ref-3] Berni M Fabbri N Fani E : Advanced Digitization for the Promotion of the Moral Values Underlying the European Union.In: V. Cappellini (Ed.): *Electronic imaging & the visual arts: EVA 2021 Florence.* Italy: Polistampa.2021;82–86.

[ref-4] Brown AL : Eteocles and the Chorus in the “ *Seven against Thebes*.” *Phoenix.* 1977;31(4):300–318. 10.2307/1087562

[ref-5] Bryman A : Social Research Methods (5th ed.).Oxford, UK: Oxford University Press.2016. Reference Source

[ref-6] Cameron HD : Studies on the Seven Against Thebes of Aeschylus.In: *Studies on the Seven Against Thebes of Aeschylus.* London: De Gruyter.1971. Reference Source

[ref-7] Charmaz K : Constructing Grounded Theory. (2nd ed.). Thousand Oaks, CA: SAGE Publications Inc.2014. Reference Source

[ref-8] Dowden K : Seven against Thebes. *Oxford Research Encyclopedia of Classics*.2016. 10.1093/acrefore/9780199381135.013.5874

[ref-9] Glaser B Strauss A : The discovery of Grounded Theory. Strategies for Qualitative Research.Chicago: Aldine Publishing.1967. Reference Source

[ref-10] Glaser B Strauss A : Discovery of Grounded Theory Strategies for Qualitative Research.New York: Routledge.2017. Reference Source

[ref-11] Grammatas T : Eisagogi stin istoria ke theoria tu theatru [Introduction to the history and theory of theatre].Athens, Greece: Exantas.2012.

[ref-12] Grammatas T : To theatro os politismiko fenomeno [Theater as a cultural phenomenon].Athens, Greece: Papazisi Press.2015. Reference Source

[ref-13] Grammatas T : Drama and Theatre in ancient Greece. A database and a spectators’ school.In: T. Grammatas & A. Papakosta (Eds.): *International Theatre Conference, Values of Ancient Greek Theatre Across Space & Time: Cultural Heritage and Memory.* Athens, Greece: Department of Primary Education, National and Kapodistrian University of Athens.2022. Reference Source

[ref-14] Grammatas T Dimaki-Zora M : Memories of heroines in memories of spectators. Mythic, dramatic and theatrical time from the ancient drama to Modern Greek Theatre.In: V. Liapis, M. Pavlou, & A. Petrides (Eds.): *Debating with the Eumenides. Aspects of the reception of Greek Tragedy in Modern Greece.* Newcastle, UK: Cambridge Scholars Publishing.2018;122–131. Reference Source

[ref-15] Hall H Roussel L : Evidence-Based Practice. An Integrative Approach to Research, Administration, and Practice. Burlington, MA: Jones & Bartlett Learning.2017. Reference Source

[ref-16] Hennink M Kaiser BN : Sample sizes for saturation in qualitative research: A systematic review of empirical tests. *Soc Sci Med.* 2022;292:114523. 10.1016/j.socscimed.2021.114523 34785096

[ref-17] Karantzouli E : The Transmission and Reception of the Values of the Ancient Greek Culture in Theater via “Digital Dialogue.”In: T. Grammatas & A. Papakosta (Eds.), *International Theatre Conference, Values of Ancient Greek Theatre Across Space & Time: Cultural Heritage and Memory.* Athens, Greece: Department of Primary Education, National and Kapodistrian University of Athens,2022. Reference Source

[ref-18] Kladaki M : I proslipsi ton evropaikon revmaton stin elliniki dramatourgia [The reception of European currents in Greek drama]. *Dromena.* 2009;2:40–43.

[ref-19] Kladaki M : 100 chronia paidiko theatro: mia istoriki anadromi tou theatrou gia paidia stin Ellada apo to 1900 os to 2000 [100 years of children’s theatre: a historical review of children’s theater in Greece from 1900 to 2000]. *Education & Theatre.* 2010a;11:7–13.

[ref-20] Kladaki M : I ennoia tis archaioellinikis mimisis os afetiriako simeio tis ennoias tou theatrou [The concept of ancient Greek imitation as a starting point for the concept of theater]. *Filologiki.* 2010b;112:38–42.

[ref-21] Kladaki M : Ta idanika ke i stoxi zois ton iroon ke ton iroidon sta theatrika erga gia pedia [The ideals and life goals of the heroes and heroines in children’s theatrical plays]. *Pedagogical Currents in the Aegean.* 2011;5:61–72.

[ref-22] Liedke H Pietrzak-Franger M : Viral theatre: Preliminary thoughts on the impact of the COVID-19 pandemic on online theatre. *Journal of Contemporary Drama in English.* 2021;9(1):128–144. 10.1515/jcde-2021-0009

[ref-23] Mason J : Qualitative Researching.(3rd ed.). London: SAGE Publications, Inc,2017. Reference Source

[ref-24] Mastrothanasis K : Psychometrikes ektimiseis tis makrochronis mnimis tou anilikou theati gia theatrikes paragoges me kallitechniki stochefsi aniliko koino [Psychometric assessments of the long-term memory of the minor spectator for theatrical productions with artistic targe.In: T. Grammatas & S. Papadopoulos (Eds.), *Time in the Theater. Theatrical Memory of a Timeless Present.* Athens, Greece: Papazisi,2021;285–303.

[ref-25] Mastrothanasis K Geladari A : Social values and children's literature: A research into the literature textbooks of Greek Primary School.In: A. Shafaei & M. Nejati (Eds.), *Annals of Language and Learning.* Boca Raton, USA: Universal Publishers - BrownWalker Press,2009;135–149. Reference Source

[ref-26] Mastrothanasis K Grammatas T : Reception of the values of the Aeschylus drama and mnemonic imprints by ancient tragedy spectators.[Data set] (V1 ed.).2022. 10.5281/zenodo.7213455 PMC10445847 37645271

[ref-27] Mastrothanasis K Kladaki M : Erevnitikos schediasmos meiktis methodologias gia tin axiologisi efarmogon Theatrou Anagnoston sti scholiki eparkeia, tin psychokoinoniki prosarmogi kai tis mathisiakes dyskolies diglosson anagnoston [A mixed-methods research design to evaluate applicatio.In: A. Sofos, M. Liarakou, M. Skoumios, & E. Fokides (Eds.), *Paidagogiki Erevna sto Aigaio, Praktika 5is Imeridas Ypopsifion Didaktoron [Pedagogical Research in the Aegean, Proceedings of the 5th Conference of Doctoral Candidates].* Rhodes: University of the Aegean,2021;143–165.

[ref-28] Mastrothanasis K Papakosta A : Values across space and time: Summary results of public research.Athens, Greece,2021. Reference Source

[ref-29] Mazzaro J : Memory in Aeschylus’ *Seven Against Thebes*. *Comparative Drama.* 1984;18(2):118–136. 10.1353/cdr.1984.0012

[ref-30] Papadopoulos S : Theatro stin ekpedefsi ke arxea elliniki skepsi [Theatre in education and ancient Greek thought].Athens, Greece: Papazisi,2021.

[ref-31] Papakosta A Mastrothanasis K Andreou A : Psychometric evaluation of recall and recognition tasks for the measurement of young spectators’ theatrical memory. *Journal of Literary Education.* 2020;3(3):177–199. 10.7203/JLE.3.14835

[ref-32] Patsalidis S : The Classics Have no Owners; Just Tenants.In T. Grammatas & A. Papakosta (Eds.), *International Theatre Conference, Values of Ancient Greek Theatre Across Space & Time: Cultural Heritage and Memory.* Athens, Greece: Department of Primary Education, National and Kapodistrian University of Athens,2022. Reference Source

[ref-33] Robinson R : Purposive Sampling.In: A. Michalos (Ed.), *Encyclopedia of Quality of Life and Well-Being Research.* Springer, Dordrecht,2014;5243–5245. 10.1007/978-94-007-0753-5_2337

[ref-34] Stehle E : Prayer and Curse in Aeschylus’ *Seven Against Thebes*. *Class Philol.* 2005;100(2):101–122. 10.1086/432841

[ref-35] Timplalexi E : Theatre and Performance Go Massively Online During the COVID-19 Pandemic: Implications and Side Effects. *Homo Virtualis.* 2020;3(2):43–54. 10.12681/homvir.25448

[ref-36] Torrance I : Aeschylus: Seven against Thebes.London & New York: Bloomsbury Academic,2007. Reference Source

[ref-37] Wong E : When Life Gives You Risk, Make Risk Theatre: Three Tragedies and Six Essays.Canada: FriesenPress,2022. Reference Source

