# Supplementary material for: Reception of the values of the Aeschylus drama and mnemonic imprints by ancient tragedy spectators
Source: Open Res Eur. 2022 Nov 3;2:124. [Version 1] doi: 10.12688/openreseurope.15179.1 (PMC10445847; doi:10.12688/openreseurope.15179.1)
Supplement: Peer review history

# Peer review: Reviewer response for version 1

**DOI:** 10.21956/openreseurope.16418.r30374

**Published:** 2022-12-06

**Authors:** Agis Marinis

**Affiliations:** 1 University of Patras, Patras, Greece

This article deals with the reception of a specific performance of ancient Greek drama by a modern Greek audience. It specifically focuses on the manner in which the humanitarian values inherent in ancient Greek tragedy are evinced through a modern performance and to what extent may the latter create a lasting impression on the audience in terms of staging and/or the values inherent in the drama. In order to respond to these questions the authors conducted research engaging a group of spectators who were requested to respond to questionnaires both immediately after watching the performance and after a six-month interval. The results of this research have been exposed in detail in this paper.

 The survey focuses specifically on a performance of Aeschylus’ *Seven against Thebes*, a drama embodying a number of key moral values and instigating reflection on issues concerning especially the issue of war and internecine strife. The authors’ research has revealed both the classification of values by the spectators, as well as the fact that this classification has not undergone significant change after six months. At the same time the authors examined whether the staging of the drama created a more lasting impression and to what extent did it support the values inherent in the text. What is of particular note is that strong mnemonic imprints were created by some compelling choices in terms of stagecraft, especially at the scene of confrontation between the two brothers.

 In sum, this is a well-thought article based on systematically conducted research, which can be considered as a significant contribution to the study of the audience reception of ancient Greek drama and the way in which it can be regarded as conveying to the modern world a number of moral values.

 A final editing of the article in order to ensure clarity of expression would be needed. Certain technical terms may also require some further elucidation.

If any, are all the source data and materials underlying the results available?

Yes

Is the work original in terms of material and argument?

Yes

Is the work clearly and cogently presented?

Yes

Is the argument persuasive and supported by evidence?

Yes

Does the research article contribute to the cultural, historical, social understanding of the field?

Yes

Does it sufficiently engage with relevant methodologies and secondary literature on the topic?

Yes

Reviewer Expertise:

Ancient Greek theatre and its reception, Classics

I confirm that I have read this submission and believe that I have an appropriate level of expertise to confirm that it is of an acceptable scientific standard.

# Peer review: Reviewer response for version 1

**DOI:** 10.21956/openreseurope.16418.r30376

**Published:** 2022-11-09

**Authors:** Avra Sidiropoulou

**Affiliations:** 1 Open University of Cyprus, Nicosia, Cyprus

This article uses a contemporary performance of Sophocles’ *Seven Against Thebes *to illustrate the universality of humanitarian values that permeate Greek tragedy. The topic is of high interest and the findings of the research significant in confirming that Greek tragedy has survived across time and space due to the timelessness of its themes and the moral questions it has perennially posited to its audiences.

 The reception of the production has been analyzed in two distinct time phases, through sample questionnaires given to a select number of participants throughout Greece. The researchers follow a solid methodology, which is clearly stated in their article. The findings of the research appear consistent with the initial premise laid out in the introduction, namely, that “Ancient drama is considered to be a link between the classic and the modern, the present and the past. Apart from any aesthetic and artistic contexts through which it forms and communicates with the modern audience through the stage as a theatrical performance, its values represent the diachronic and the universal in cultural creation”.

 The authors are clear about the intended outcome of the research, the methodology they employed during the six-month long study of the audience’s responses to the given production and present their argument persuasively and through specific examples. The data concerning the research has been made available in its entirety.

 One thing to note is that more careful editing for clarity of syntax and expression is due, as the article contains several verbal repetitions and oversights and (minor) syntactical errors that might muddle meaning. Equally, some terms, such as “appropriation” need theoretical reframing and clarification.

If any, are all the source data and materials underlying the results available?

Yes

Is the work original in terms of material and argument?

Yes

Is the work clearly and cogently presented?

Yes

Is the argument persuasive and supported by evidence?

Yes

Does the research article contribute to the cultural, historical, social understanding of the field?

Yes

Does it sufficiently engage with relevant methodologies and secondary literature on the topic?

Yes

Reviewer Expertise:

Theory and methodology of directing; contemporary theatre practice; adaptation and ethics.

I confirm that I have read this submission and believe that I have an appropriate level of expertise to confirm that it is of an acceptable scientific standard.
